# Validation and Variation of Upper Layer Thickness in South China Sea from Satellite Altimeter Data

**DOI:** 10.3390/s8063802

**Published:** 2008-06-06

**Authors:** Chun-Yi Lin, Chung-Ru Ho, Zhe-Wen Zheng, Nan-Jung Kuo

**Affiliations:** Department of Marine Environmental Informatics, National Taiwan Ocean University, Keelung, Taiwan; E-mail: D94810002@mail.ntou.edu.tw

**Keywords:** Upper layer thickness, satellite altimeter, South China Sea, El Niño

## Abstract

Satellite altimeter data from 1993 to 2005 has been used to analyze the seasonal variation and the interannual variability of upper layer thickness (ULT) in the South China Sea (SCS). Base on in-situ measurements, the ULT is defined as the thickness from the sea surface to the depth of 16°C isotherm which is used to validate the result derived from satellite altimeter data. In comparison with altimeter and in-situ derived ULTs yields a correlation coefficient of 0.92 with a slope of 0.95 and an intercept of 6 m. The basin averaged ULT derived from altimeter is 160 m in winter and 171 m in summer which is similar to the in-situ measurements of 159 m in winter and 175 m in summer. Both results also show similar spatial patterns. It suggests that the sea surface height data derived from satellite sensors are usable for study the variation of ULT in the semi-closed SCS. Furthermore, we also use satellite derived ULT to detect the development of eddy. Interannual variability of two meso-scale cyclonic eddies and one anticyclonic eddy are strongly influenced by El Niño events. In most cases, there are highly positive correlations between ULT and sea surface temperature except the periods of El Niño. During the onset of El Niño event, ULT is deeper when sea surface temperature is lower.

## 1. Introduction

The South China Sea (SCS) is the largest marginal sea at the westernmost side of the tropical Pacific Ocean. It is surrounding an area from Singapore to the Strait of Taiwan of around 3,500,000 km^2^. The water body of SCS connects with the East China Sea, the Pacific Ocean, and the Indian Ocean mainly through the Taiwan Strait, the Luzon Strait, and the Strait of Malacca, respectively. The bottom topography of the sea is characterized by two extended continental shelves on the northern and the southern sides and a deep basin with a maximum depth of 5000 m situated in the central-eastern portion. The deep basin occupies 44% of the total area of the SCS. The hydrology of the SCS is deeply influenced by the Southeast Asian monsoon system. Dynamic characteristics in the SCS have been investigated by previous studies [1-4] can be primarily summarized as: (1) the circulation driven by seasonal monsoon induce basically a cyclonic gyre in winter and an anticyclonic gyre in summer. (2) Cyclonic eddy located at off west Luzon in winter and a dipole structure with a coastal jet off east Vietnam in summer. (3) El Niño events affect the wind field and influence the circulation eventually. As a part of the western Pacific warm pool, SCS plays a major role in hydrology and climatology to the surrounding areas [6]. The sea surface temperature (SST) is a major signature of upper ocean thermal processes. It is affected by upper layer variability to a large extent. On the other hand, the sea surface height (SSH) as an integrated response of the entire water column primarily reflects ocean dynamic variability. Thus, assuming a constant salinity over the upper layer in the SCS, the change in SSH should follow the heat input. In other words, SSH may record the thermal input into the ocean in ignoring the dynamic processes.

Satellite altimeter data has provided an opportunity to derive a good quality SSH dataset [8]. The estimates of upper layer thickness (ULT) from satellite altimeter data have also been studied since 1990s. Shay et al. [5] using satellite altimeter and SST data as input to a two-layer reduced gravity model, one can obtain a first order approximation of the upper layer thermal structure. Goni et al. [9] estimated the ULT in the southwestern Atlantic Ocean from Geosat altimeter data and fitted fairly well to the results of inverted echo sounder. Garzoli et al. [10] used the altimeter data derived ULT to monitor the upper layer transport in the southeastern Atlantic Ocean. Pun et al. [11] used altimeter derived ULT to improve typhoon intensity forecast in the Western North Pacific Ocean. The spatial and temporal variability of the split of Kuroshio extension was also studied by using the altimeter data derived ULT [12]. A further application of altimeter data derived ULT is to study the heat storage in the upper layer combined with sea surface temperature [13-16].

**Figure 1.**
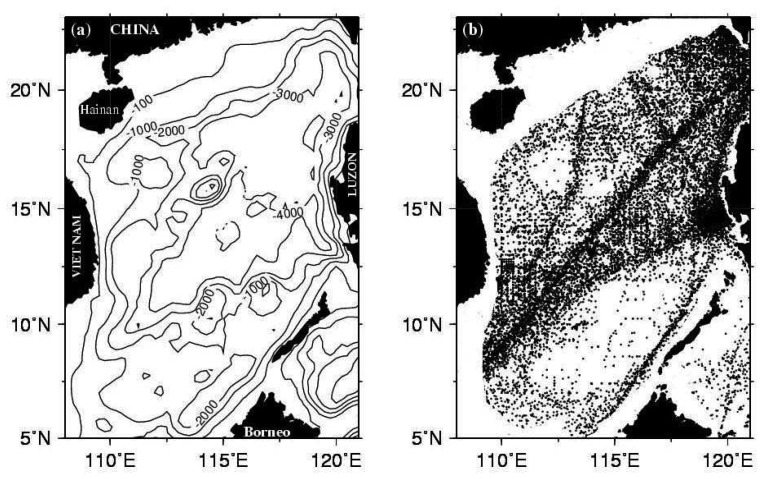
(a) Bottom topography (b) in-situ measurements station distribution from 1980 to 2005 in the South China Sea. Region with water depth shallower than 200 m is stripped. Numbers on isobaths show the water depths in meter.

The applications of satellite derived ULT are most in the open oceans; however, in this study we validate the usability of this estimation in a semi-closed sea. The spatial and temporal variability of ULT is also investigated. The study area is from 5°N to 23°N and from 108°E to 121°E covering most of the deep basin of the SCS as shown in Figure 1a. This paper is organized as follows. In section 2, the data and data processing are introduced. Section 3 provides a two two-layer reduced gravity ocean model for estimating ULT. The ULT derived from satellite altimeter data is validated in section 4. Seasonal and interannual variability of ULT in the SCS and the ULT influenced by SST are discussed in section 5. Finally, a summary is given in section 6.

## 2. Data and Data Processing

TOPEX/Poseidon (T/P) and Jason-1 altimeter data used in this study is processed and provided by the National Aeronautics and Space Administration (NASA) Physical Oceanography Distributed Active Archive Center (PO.DAAC) at the Jet Propulsion Laboratory (JPL), California Institute of Technology. The precision of the T/P altimeter system is on the order of 4 cm for each measurement [8, 18, 19]. The Jason-1 satellite was launched to extend the long-term success of T/P oceanographic mission. This provides an extended continuous time series of high-accuracy measurements of the ocean surface topography from which scientists can detect the Earth's climate change. The accuracy of sea level anomalies can be as high as 2.5 cm or better [20]. The data have been corrected for the effects of the following: troposphere, ionosphere, inverse barometer, sea state bias, and tides. The T/P and Jason-1 altimeter data along the tracks within our study area from 1993 to 2005 constitutes a baseline for this study. We computed the collinear residual SSH to remove the unknown geoid, which is time invariant, and removed a nine-year mean from 1993 to 2001. The products are defined as sea surface height anomaly (SSHA) data [21]. Accordingly, the average SSH is computed to 1° latitude by 1° longitude grid with one month resolution after interpolations.

The in-situ hydrographic data (World Ocean Database 2005) used in this study are provided by the National Oceanographic Data Center (NODC) of National Oceanic and Atmospheric Administration (NOAA). The data consist of hydrographic parameters collected from hydrographic casts including conductivity-temperature-depth (CTD) probes, bottle low resolution CTD, mechanical expendable (XBT), digital bathythermographs, profiling float, autonomous pinniped bathythermograph data, drifting buoy data, moored buoy data and undulating oceanographic recorder data [17]. We process the data to generate monthly and 2° latitude by 2° longitude grid data sets. The standard deviation check is used to remove the outliers. This means that the data points being more than three standard deviations apart from the mean are removed. These data points are considered as being affected by transient effects of waves, strong winds, or other dynamic processes. After data processing, about thirty million hydrographic profiles from January 1980 through December 2005 are obtained. Their distribution is shown in Figure 1b. The main limitation of this kind of in-situ measurements is its uneven distribution in time and space. Certain periods and areas are over sampled, while others are under. Even so, the total number of data is enough for the climatologic analysis.

**Figure 2.**
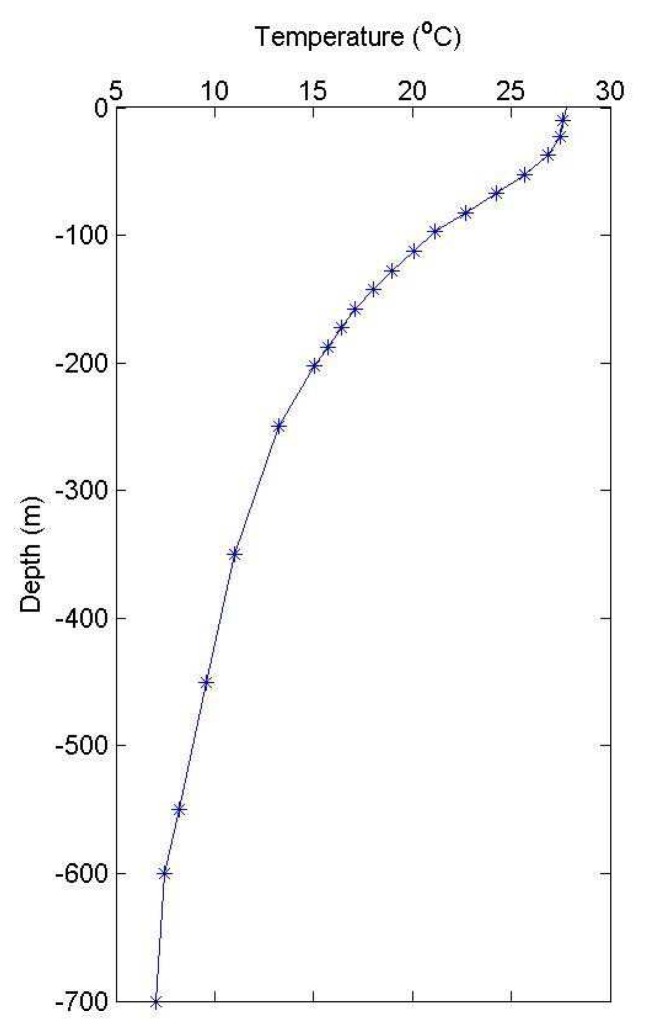
The 20-year mean climatological temperature profile in the SCS.

**Figure 3.**
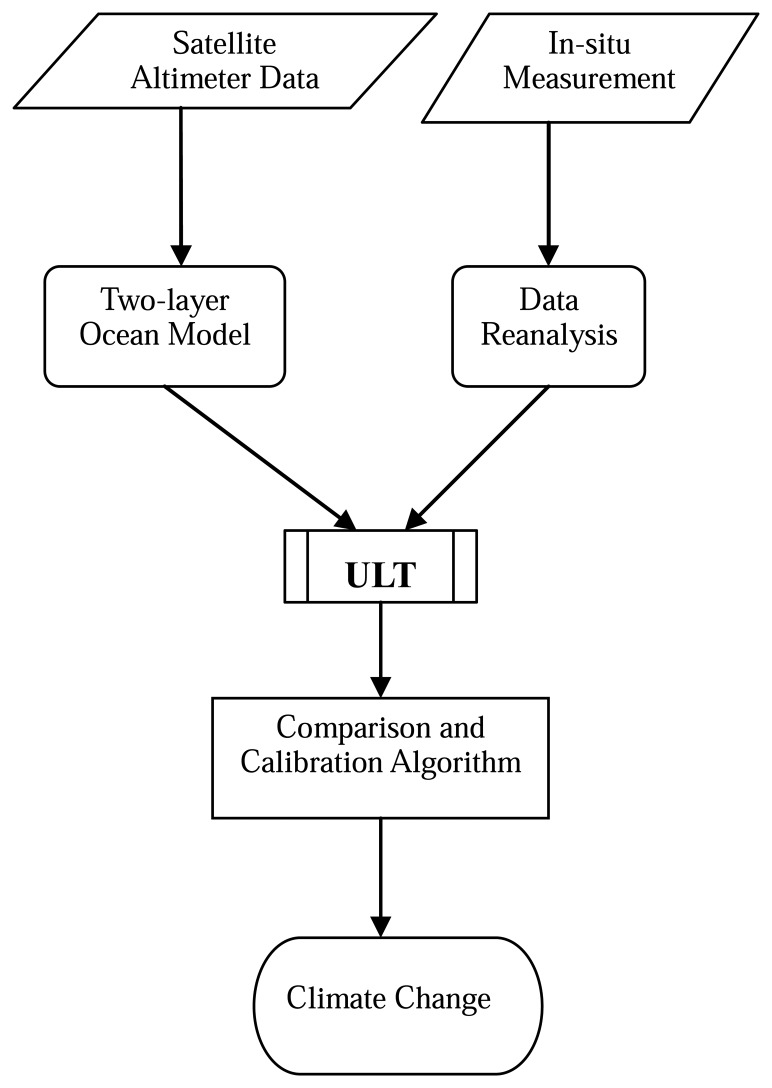
Flowchart describes the process of ULT estimated.

The upper layer is the main heat storage layer in the ocean, which has important effects on the ocean circulation and climate. In this study, the upper layer is defined as the layer from the sea surface to the bottom of thermocline. The definition of thermocline is a zone with a rapid change in temperature with depth. Although the definition of thermocline is clear, in practice its depth is difficult to be determined. For the tropic oceans, previous investigators used the depth of a certain isotherm as the thermocline depth. For example, Worthington [22] used the depth of the 18°C isotherm and Wang et al. [23] used the depth of the 20°C isotherm as the depth of thermocline. In their cases, however, the depths of either 18°C or 20°C isotherm only lie within the center of thermocline, not the bottom of thermocline. In our case, the mean temperature profile that is averaged from each temperature profile from 1980 to 2005 is shown in Figure 2. The vertical structure of the temperature profile averaged from 1980 to 2005 in SCS show that the largest vertical temperature gradients are located between 14°C and 20°C. This temperature profile is similar to that in Grey et al. [24], who adapted the depth of the 16°C isotherm as the depth of bottom thermocline. Based upon temperature variability from hydrographic measurements, the choice of the 16°C isotherm depth is deemed appropriate for the assumed two-layer ocean in this analysis. Figure 3 summaries the process of data analysis and using in-situ data to calculate the ULT.

## 3. Two-Layer Reduced Gravity Ocean Model

The relationship between SSH and the mass field of the ocean allows these two parameters to be used within a two-layer reduced gravity ocean model to monitor ULT. Although there are other factors linking to SSH, such as salinity changes, salinity changes are smaller than thermal effects. Seasonal changes in the surface salinity due to fresh water flux are small over most of oceans, expect near river outlets and regions of upwelling. The annual surface salinity amplitude is less than 0.2 psu (practical salinity unit) in the SCS [7]. Assuming a mixed layer depth of 100 m, a constant salinity change of 0.2 over this depth, what's more, salinity variability decrease with depth, the sea level amplitude would be 1.5 cm [25, 26]. Sato et al. [30] and Polito et al. [31] have shown that the sea level effects due to salinity changes can be corrected by haline correction (*η_h_*). The *η_h_* (*x*, *y*,*t*) is estimated by integral of the product of the climatological haline contraction coefficient, *β*, and the salinity anomaly Δ*S* (residual after subtracting the annual mean) profiles from the surface to a depth h.

(1) $$\eta_{h}=\int_{-h}^{0}\beta \Delta \text{Sdz}.$$

In order to realize the haline effects we chose the area in the SCS defined inside a rectangular box from 110°E to 117°E and from 10°N to 17°N. By using Eq. (1), it is estimated that the contribution of the salinity fluctuation to SSH variation is about 1 to 3 cm based on World Ocean Database 2005 salinity dataset. Comparing the SSHA in the SCS, the effect of salinity on the sea level change is very small, thus a reasonable approximation of the SSH change in the water column can be derived from the temperature change.

It is assumed here that major variability is due to changes in the depth of the thermocline and in barotropic origin. Figure 3 summaries the process of using SSH and in-situ data to calculate the ULT. According to the results by previous investigators, the ULT can be estimated using satellite altimeter-derived SSH [9, 10, 12, 16] by

(2) $$H(x,y,t)=H_{a}(x,y)+\frac{g^{\prime }(x,y)}{g(x,y)}\times \eta (x,y,t)+C(x,y),$$

and

(3) $$g^{\prime }(x,y)=g(x,y)\times \frac{\rho_{2}(x,y)-\rho_{1}(x,y)}{\rho_{2}}.$$

where *H*(*x*, *y*,*t*) is the altimeter-derived ULT, *x* and *y* constitute a two-dimensional horizontal coordinate system, *t* is the time, *H_a_* (*x*, *y*) is the mean climatological ULT, *g*(*x*,*t*) is the gravity acceleration and is treated as a constant, *g′*(*x*, *y*) is the reduced gravity acceleration, *ρ*_1_ and *ρ*_2_ are the mean densities of upper layer and lower layer, respectively, *η*(*x*, *y*,*t*) is the altimeter-derived SSHA, and *C*(*x*, *y*) is proportional to the barotropic contribution. *C*(*x*, *y*) can be estimated if simultaneous observations of SSH and thermocline depth are available [9, 12].

Thus, to compute the local ULT, the mean ULT, the densities of upper layer and lower layer, as well as local SSHA must be known (Figure 3). In this study, the mean ULT and densities of upper layer and lower layer are derived from in-situ measurements. According to aforementioned section, the ULT is defined as the thickness from sea surface to the isotherm of 16°C. Therefore, we interpolate each temperature profile of the in-situ data set to obtain the depth of the 16°C isotherm and then averaged them to two-degree grid. The averaged depth is treated as the mean ULT. Because the SCS encompasses a numbers of different regions; therefore, we calculates the mean ULT in the SCS on central SCS, exclusive the regions with water depth shallow than 200m, the Taiwan Straits and semi-enclosed Sulu Sea. The mean reduced gravity acceleration is computed from the densities of upper layer and lower layer. We average the density of each profile from surface to the depth of the 16°C isotherm and that from the depth of the 16°C isotherm to 1000 m to obtain the mean densities of upper layer and lower layer, respectively. The reduced gravity acceleration at each two-degree grid is then derived. The barotropic contribution is estimated from the overlay period of SSHA and ULT, i.e., 1993 ∼ 2005. Finally, substituting the satellite derived SSHA, the mean ULT, the reduced gravity acceleration, and the barotropic component into Eq. (2) yields the local ULT. In this study, we focus the ULT variability on the central SCS.

## 4. Validation

**Figure 4.**
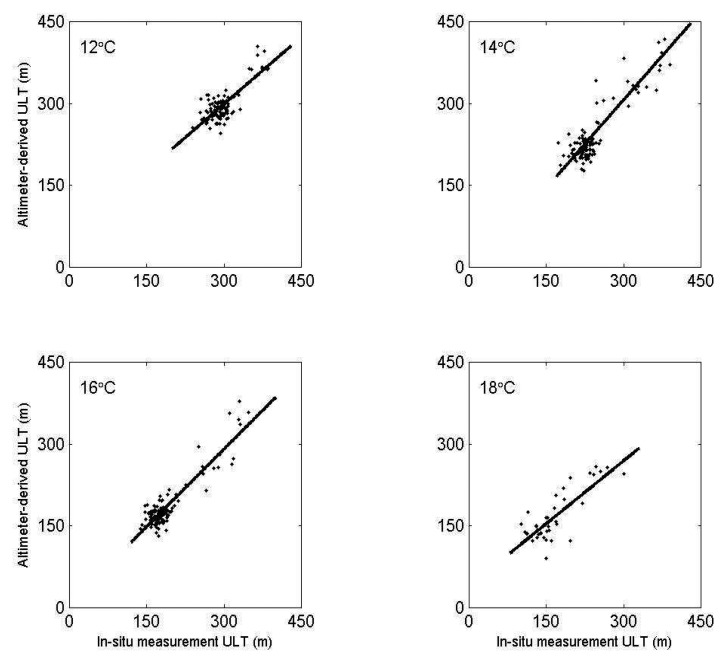
The scatter plots of altimeter derived ULT and in-situ measurements of the depth of 12°C, 14°C, 16°C, and 18°C isotherms. The best line fit is represented by the solid line.

**Figure 5.**
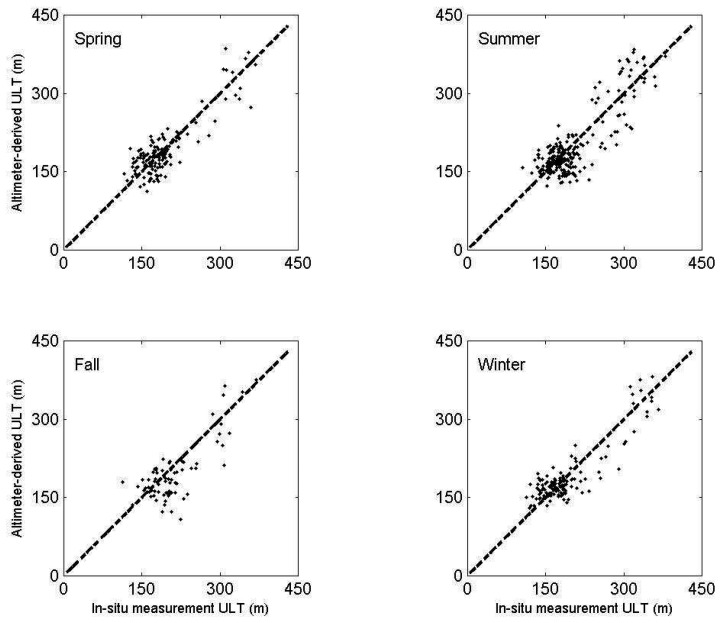
The scatter plots of altimeter derived ULT and in-situ measurements of the depth of 16°C isotherm in spring, summer, fall, and winter. The line y=x is represented by the dashed line.

T/P and Jason altimeter data from 1993 to 2005 is used to derive ULT in the SCS by Eqs. (2) and (3). The calculated ULT is validated with the in-situ measurements. In order to test if the depth of the 16°C isotherm is suitable for defining the ULT in the SCS, we also use the depths of 12°C, 14°C and 18°C isotherms for comparison. Figure 4 shows comparisons of ULT derived from altimeter data with the results from in-situ measurements using the depth of 12°C, 14°C, 16°C, and 18°C isotherms, respectively. The satellite derived ULT are in good agreement with those calculated from four kinds of isotherms. However, one can see that the best result is by using the definition of the depth of the 16°C isotherm as the thickness of upper layer (Table 1). The slope and intercept which are calculated by linear regression at the significant 95% confidence level between SSH derived ULT and in-situ measurements are 0.92 and 6 m, respectively with a correlation coefficient of 0.95. In order to understand the accuracy of estimated ULT, we further compare the ULT derived between altimeter data and in-situ measurements in four seasons (Figure 5). The correlation coefficient, the root mean square (RMS), the mean absolute percentage error (MAPE), and bias are calculated to validate the accuracy of satellite derived ULT. Table 2 shows high correlation coefficient and low RMS, MAPE and bias, although results in fall are worse than others. The satellite derived ULT is not influenced by seasonal change in the SCS. This indicates that the two-layer reduced gravity ocean model may be suitable for deriving ULT from SSH in this semi-closed sea. However, among averaged ULT derived from two-degree grid data reduced gravity model, it may be good for large-scale climate researches in our study but definitely is misleading for meso-scale features such as eddies and internal waves. On the other hand, we believed that the two-layer reduced gravity ocean model has potential to detect meso-scale features with higher temporal and spatial resolutions by merged altimeter data of multi-sources.

## 5. Variation

**Figure 6.**
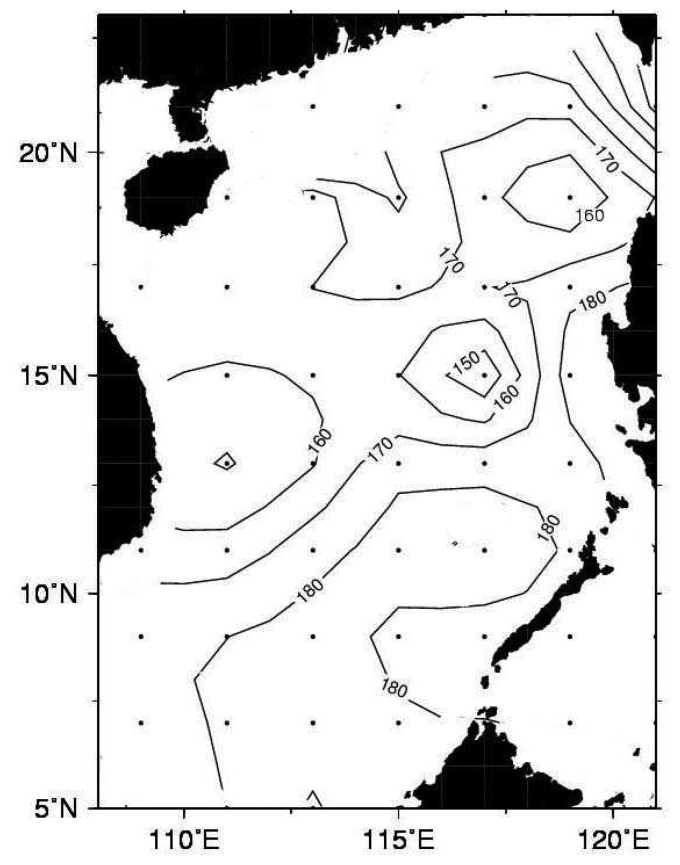
The 26-year mean ULT distribution derived from in-situ measurements (1980-2005). Dots on the figure are the grid points of data.

**Figure 7.**
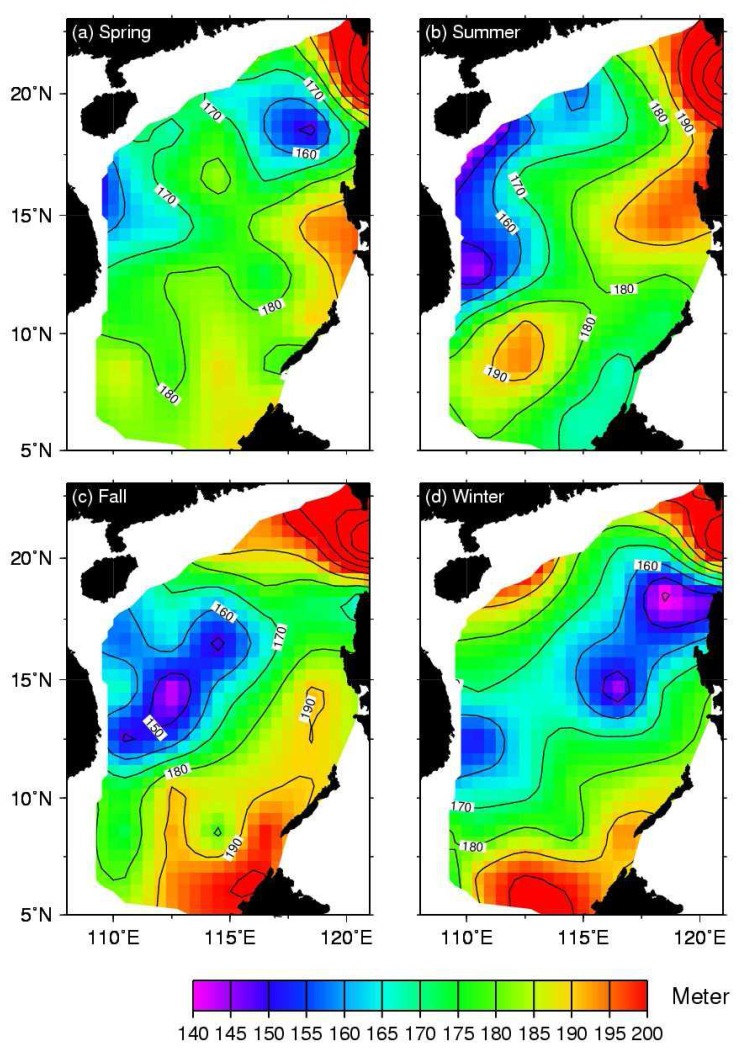
Seasonal mean ULT derived in-situ measurements from 1980 to 2005.

Using the depth of the 16°C isotherm, the distribution of mean ULT from 1980 to 2005 in the SCS is calculated. The result is shown in Figure 6. The mean ULT is about 168 m. Thick ULT is toward the Luzon Strait. Furthermore, one also can see that there is a thicker ULT area at 15°N, 116°E and a thinner ULT area at 18°N, 118°E. From the point of view of specific volume, a thinner (thicker) ULT would cause a lower (higher) SSH. Therefore, it seemed that there is a cold eddy-like feature around 18°N, 118°E and a warm eddy-like feature around 15°N, 116°E. These eddy-like phenomena have been reported by previous studies [2, 27, 28, 29]. However, these eddy-like features have seasonal variation. Figure 7 shows the distribution of in-situ seasonal mean ULT in the SCS. In order to exhibit the similarity of dominant features on the images, we divide a year in SCS in to four seasons with the definition of Ho et al. [21], that is, winter: November, December, January, and February; spring: March and Arial; summer: May, June, July, August; and fall: September and October. One can see that the cold eddy-like feature shows up near northwest Luzon in winter and spring, and the cold eddy-like feature decays after April. Chu [2], Qu [29], and Ho et al. [21] gave similar results. The ULT at 18°N, 118°E is about 142 m in winter and 182 m in summer. From Figure 7, one can see that the Vietnam coastal upwelling causes the ULT in summer to be thin [28, 33, 34]. At the same time, a coastal jet over the shelf in the southern part of Vietnam forms an anticyclonic eddy [32]. This warm eddy-like feature exhibits clearly around 9°N in summer (Figure 7). Moreover, the averaged ULT of the all basin derived from altimeter data is about 160 m in winter, 170 m in spring, 171m in summer, and 164 m in fall. Its seasonal change is about 11 m. As shown in Table 2, the RMS of ULT compared between altimeter and in-situ measurements is about 30 m. The seasonal difference of 11 m is within the uncertainty of ULT. However, the bias is as low as 5 m. It indicates that we could minimize the random error and improve the accuracy by taking the average of the seasonal ULT variations. Besides, they are also comparable to the in-situ measurements with 159 m in winter, 171 m in spring, 175 m in summer, and 170 m in fall as well as a seasonal difference of 16 m. The deviation of 4 m from altimeter and in-situ measurements suggests that the seasonal variation estimated by altimeter data is reliable.

**Figure 8.**
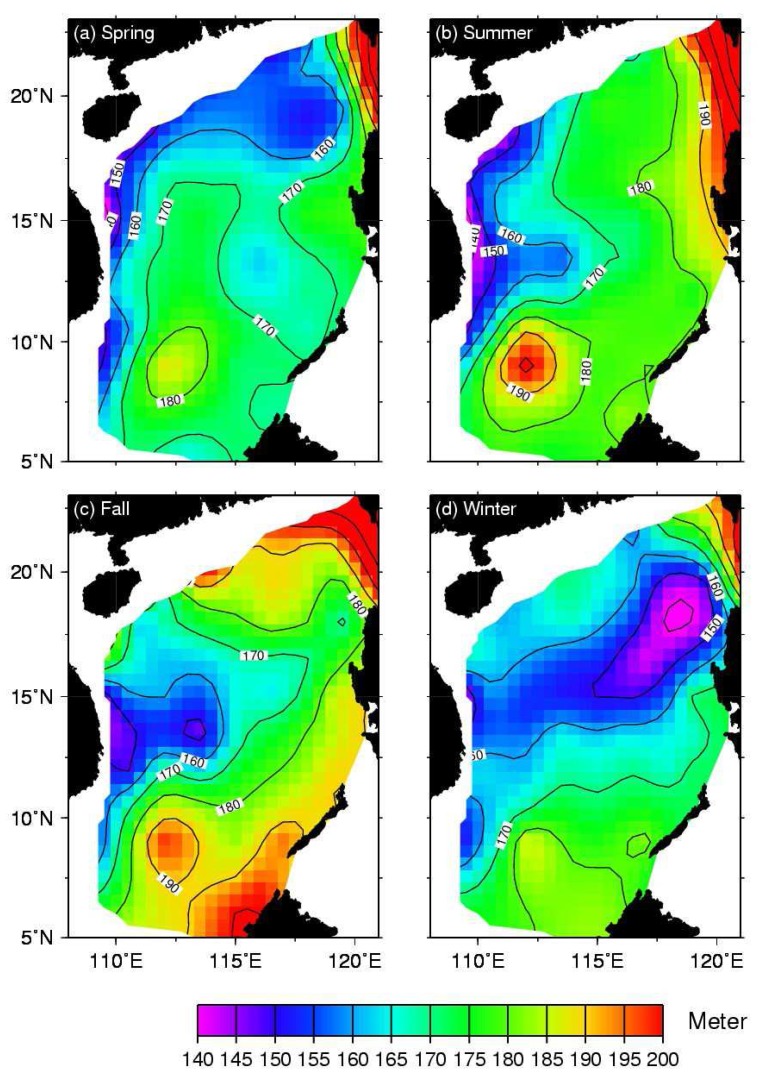
Seasonal mean ULT derived from T/P and Jason altimeter data from 1993 to 2005.

**Figure 9.**
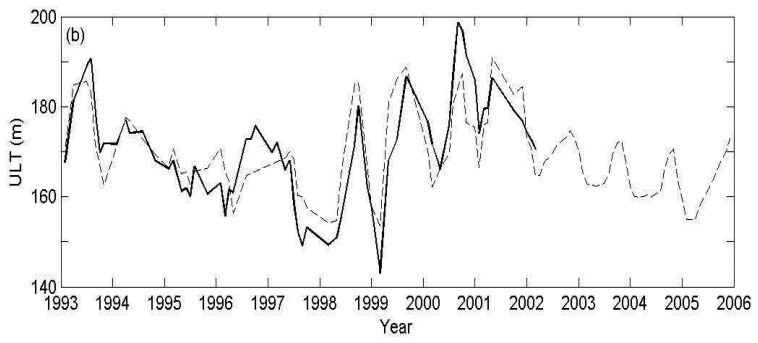
Time series of monthly ULT averaged over SCS (solid line: derived from in-situ measurements; dashed line: derived from altimeter SSHA data).

The seasonal distribution of ULT derived from T/P and Jason altimeter data from 1993 is shown in Figure 8. The distribution of the ULT derived from SSH is similar to that derived from in-situ measurements in the basin (Figure 7) except the area near the southern Taiwan Strait. The cold eddylike feature at 18°N, 118°E northwest of Luzon and at 13°N, 111°E east of Vietnam and the warm eddy-like feature at 9°N, 112°E southeast of Vietnam as shown in Figure 7 are also presented in the SSH derived ULT by two-layer reduced gravity ocean model. The differences in the southern Taiwan Strait are probably caused by the imprecision of SSH data in the shallow water area. A plot of combined monthly mean ULT derived from in-situ measurements (1993-2002) averaged over the basin of SCS and satellite altimeter data (1993-2005) is shown in Figure 9. The ULT derived from in-situ data is missing after 2002 because the data are only located near Luzon coast. One can see a good match of ULT during the overlap period with a correlation coefficient of 0.81 and a RMS of 7 m. This provides an opportunity to extend the time series of ULT when there is no in-situ measurement but satellite data is available and gets synchronous ULT distribution in the whole basin.

**Figure 10.**
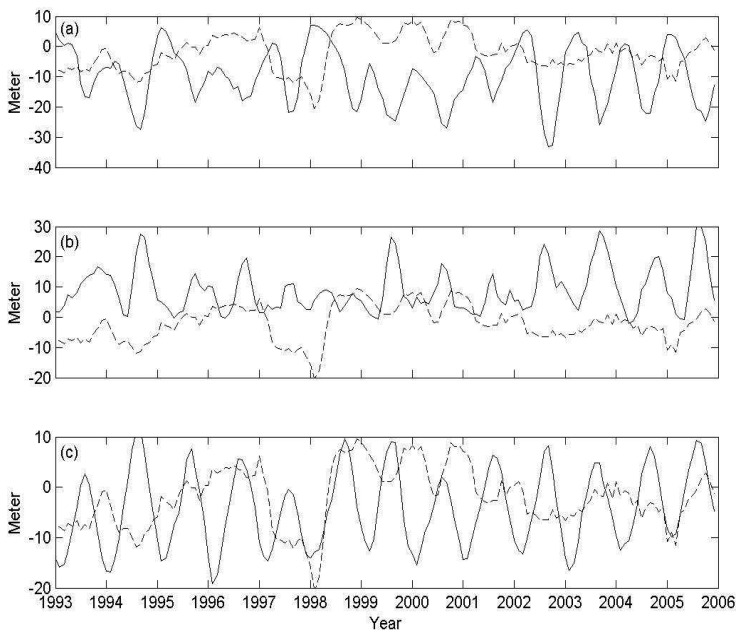
(a) Vietnam cyclonic eddy index (solid line), (b) Vietnam anticyclonic index (solid line), and (c) Luzon cyclonic index (solid line). Dashed line is SOI index.

In order to investigate interannual eddy-like feature in the SCS, we define a ULT index as the difference between ULT averaged in the eddy-like region and near surrounding areas. Figure 10 shows the ULT index of off Vietnam cyclonic eddy, anticyclonic eddy and off west Luzon eddy. It reveals the eddy intensities, with interannual and seasonal variations in the SCS. The peak values in Figure 10a and Figure 10b present the upwelling and the downwelling phenomena, respectively. Characteristics of interannual variability appear in the high and low values during summers of 1994, 2002-2003, and 2004-2005 which are primarily in good agreement with El Niño events. The enhancements of the dipole feature are attributed to be associated with the strengthening of southwest monsoon and formation of the eastward jet during El Niño years. However, the ULT doesn't show special anomalous during 1997-1998 El Niño events because the whole SCS was influenced by this enormous El Niño events. Figure 10c shows the upwelling pattern in winter, but it is weaker during 1998-2001 winter when is the La Niña period. The extra-shallow ULT off Luzon in 1998 winter is found. The west Pacific water which intrudes the northern SCS diminishes the strength of cyclonic eddy and shallows the ULT off west Luzon during La Niña events [35]. Wu and Chang [3] also suggested the weakening upwelling off Luzon in winter 1998 and explained it with abnormal warming starting from summer 1998.

**Figure 11.**
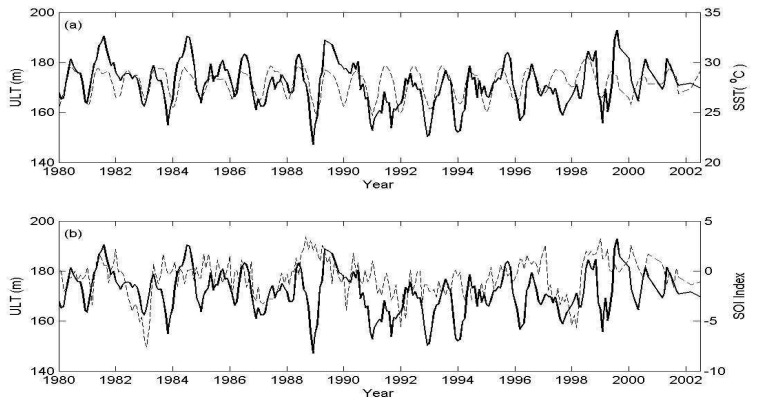
(a) A comparison of time series of monthly ULT (solid line) and SST (dashed line) averaged over SCS. (b) ULT (solid line) and SOI index (dashed line).

SST is an important signature of upper ocean thermal processes. From the point of view of specific volume, increasing SST causes specific volume increase and also SSH and ULT increase. Figure 11a shows a time series of monthly ULT (solid line) and SST (dashed line) averaged over SCS. Figure 11b shows comparison between the ULT (solid line) and the ULT Southern Oscillation Index (SOI) (dashed line). From the time series of monthly mean ULT and SST (Figure 11a), one can find that both are consistent except the periods of El Niño. In general, ULT is deeper in summer and shallower in winter during normal and La Niña years, but ULT is deeper in winter and shallower in summer during El Niño years. The seasonal variation can be as high as 50 m during the 1988-1989 La Niña event. However, the seasonal variation is low to 9 m influenced by the 1986-1987 El Niño event. Although SST can show warming pattern and cooling pattern in SCS, the ULT presents larger variations. It suggests that the ULT can reflect more realistic thermal structure in the ocean than that from SST. To further examine the relationship between SST and ULT, the correlation coefficient of monthly averaged ULT and SST from January 1980 to December 2005 was computed. The correlation coefficient is 0.49, however the coefficient can be increased to 0.70 when El Niño periods are excluded.

## 6. Summary

Variations of monthly mean ULT in the SCS are derived from in-situ measurements from 1980 to 2005 and satellite altimeter data from 1993 to 2005. The depth of the 16°C isotherm is chosen as definition of the thickness of upper layer. Comparison of the ULT derived from satellite altimeter data with that derived from in-situ measurements yields a correlation coefficient of 0.92 with the slope of 0.95 and the intercept of 6 m. It indicates that the ULT can be derived from satellite altimeter data by the two-layer reduced gravity ocean model in the SCS. However, the two-degree grid ULT data may be good for large-scale climate study but be not suitable for meso-scale features. The basin averaged ULT derived from in-situ measurements and satellite altimeter data is about 168 m and 165 m in the SCS with a seasonal variation of 17 m and 11 m, respectively. The ULT is thin in winter and thick in summer. Furthermore, seasonal mean ULT shows similar spatial patterns between in-situ measurements and satellite altimeter data. We also use satellite derived ULT to detect the development of eddy. There are two meso-scale cyclonic eddies and one anticyclonic eddy found in the SCS. The ULT is also highly correlated with the sea surface temperature in the study area except the periods of El Niño. During normal and La Niña years, ULT is deeper when SST is higher. However, during the onset of El Niño event, a reverse result is found, that is, ULT is deeper when SST is lower. From this study, we suggest that the SSH and SST data derived from satellite sensors are usable for study the variation of ULT in the SCS.

## Figures and Tables

**Table 1.** Regression analyses between in-situ and SSHA derived ULT.

Temperature of ULT depth (°C)	Correlation coefficient	slop	Intercept (m)
12	0.78	0.82	53
14	0.90	1.07	-17
16	0.92	0.95	6
18	0.83	0.78	38

**Table 2.** Comparison between altimeter derived ULT and in-situ measurements of the depth of 16°C.

Season	Correlation coefficient	RMS (m)	MAPE (%)	Bias (m)
Spring	0.88	28	11	1
Summer	0.85	31	12	-3
Fall	0.79	41	14	10
Winter	0.90	26	11	-3
